# Clinical outcomes of uterine artery embolization in different Kishi classification types of adenomyosis

**DOI:** 10.3389/fmed.2026.1922274

**Published:** 2026-08-26

**Authors:** Wen-Jiang Wei, Song Chen, Zuo-Fu Tian, Cheng-Jiang Xiao, Bi-Ting Zhong, Zhu-Ting Fang

**Affiliations:** 1 Department of Oncology and Vascular Interventional Therapy, Fujian Key Laboratory of Translational Cancer Medicine, Clinical Oncology School of Fujian Medical University, Fujian Cancer Hospital (Fujian Branch of Fudan University Shanghai Cancer Center), Fuzhou, Fujian, China; 2 Interventional Vascular Department, The Affiliated Guangdong Second Provincial General Hospital of Jinan University, Guangzhou, Guangdong, China; 3 Department of Interventional Radiology, The First Affiliated Hospital of Sun Yat-sen University, Guangzhou, China; 4 Department of Gynecology, The Affiliated Guangdong Second Provincial General Hospital of Jinan University, Guangzhou, Guangdong, China

**Keywords:** adenomyosis, CA125, health-related quality of life, Kishi classification, numerical rating scale, ovarian endometrioma, symptom severity scale, uterine artery embolization

## Abstract

**Objective:**

Uterine artery embolization (UAE) is an effective minimally invasive treatment for adenomyosis (AM). This study aimed to evaluate the clinical outcomes of UAE in patients with different AM subtypes according to the Kishi classification and to investigate the predictive value of Kishi classification for treatment efficacy and long-term recurrence.

**Methods:**

A total of 467 patients with AM who underwent UAE were retrospectively enrolled and classified into four subtypes (types I–IV) according to the Kishi classification. Baseline demographic and clinical characteristics, as well as preoperative and 3-month postoperative Symptom Severity Scale (SSS), Health-Related Quality of Life (HRQOL), and Numerical Rating Scale (NRS) scores, were collected and analyzed. Multivariable logistic regression was performed to identify factors associated with clinical response and 5-year efficacy after UAE.

**Results:**

Significant differences were observed among the four Kishi subtypes in terms of age, number of miscarriages, uterine volume, hemoglobin level, serum cancer antigen 125 (CA125) level, and the presence of concomitant ovarian endometrioma (all *p* < 0.05). Type II patients exhibited the lowest preoperative SSS scores. At 3 months after UAE, SSS and NRS scores significantly decreased and HRQOL scores significantly improved in all subtypes. Type I patients demonstrated the lowest postoperative SSS and NRS scores and achieved significantly higher rates of dysmenorrhea relief and clinical response than patients with type II and type IV disease. Type II patients had significantly lower clinical response rates than those with types I, III and IV. Type II and III patients experienced a higher rate of 5-year recurrence following UAE than type I and type IV patients. Multivariable analysis identified Kishi classification as an independent predictor of both clinical response and 5-year efficacy after UAE.

**Conclusion:**

Kishi classification is an independent predictor of clinical efficacy and long-term recurrence following UAE in patients with adenomyosis. Among the four subtypes, type I is associated with the most favorable therapeutic outcomes, whereas type II carries the highest risk of recurrence. These findings suggest that Kishi classification may serve as a useful tool for patient stratification and prognostic assessment before UAE.

## Introduction

Adenomyosis (AM) is a chronic estrogen-dependent gynecologic disorder characterized by the presence of ectopic endometrial glands and stroma within the myometrium, leading to progressive uterine enlargement, abnormal uterine bleeding, dysmenorrhea, chronic pelvic pain, and infertility (1). Recent epidemiological studies suggest that AM affects up to 20–35% of women of reproductive age and substantially impairs quality of life, work productivity, and reproductive outcomes (2). Despite its high prevalence and clinical burden, optimal management of AM remains challenging because of its heterogeneous pathogenesis, variable clinical manifestations, and diverse imaging phenotypes (3).

Current treatment strategies for AM include hormonal therapies, conservative surgery, and hysterectomy (4). Hormonal treatments, including gonadotropin-releasing hormone (GnRH) agonists and the levonorgestrel-releasing intrauterine system (LNG-IUS), can effectively alleviate symptoms but are frequently associated with adverse effects, symptom recurrence after treatment discontinuation, and limited efficacy in patients with extensive disease (5). Although hysterectomy remains the definitive treatment, it is unsuitable for women seeking uterine preservation (6). Conservative adenomyomectomy may improve symptoms in selected patients; however, concerns regarding surgical complexity, recurrence, and potential uterine rupture during subsequent pregnancy limit its widespread application (7). Consequently, there is an increasing need for effective uterus-sparing therapies that provide durable symptom control while preserving reproductive potential.

Uterine artery embolization (UAE) has emerged as an established minimally invasive alternative to surgery for symptomatic AM (8). By selectively occluding the uterine arterial supply, UAE induces ischemic necrosis of adenomyotic lesions and has demonstrated favorable outcomes in reducing dysmenorrhea, heavy menstrual bleeding, and uterine volume while improving health-related quality of life (9). Accumulating evidence from prospective studies and systematic reviews has confirmed the safety and long-term efficacy of UAE, with clinical outcomes comparable to those achieved with more invasive surgical approaches in appropriately selected patients (10). Nevertheless, considerable heterogeneity in treatment response and recurrence remains a major clinical challenge, highlighting the need for reliable imaging biomarkers that can facilitate patient selection and prognostic stratification.

Magnetic resonance imaging (MRI) plays a pivotal role in the diagnosis and characterization of AM (11). Among several imaging-based classification systems, the Kishi classification, first proposed in 2012, has gained increasing recognition because it reflects potential differences in disease origin and pathophysiology. Based on the predominant location of adenomyotic lesions within the uterine wall, AM is categorized into intrinsic (type I), extrinsic (type II), intramural (type III), and indeterminate (type IV) subtypes (12). Emerging evidence suggests that these subtypes exhibit distinct clinical and biological characteristics. Intrinsic AM is frequently associated with uterine injury and prior intrauterine procedures, whereas extrinsic AM shows a strong association with pelvic endometriosis and deep infiltrating endometriosis (13). Furthermore, significant differences in symptom profiles, reproductive outcomes, lesion distribution, and treatment responses have been reported among Kishi subtypes (14). Recent studies have demonstrated that imaging-based subtyping may influence therapeutic outcomes following uterus-preserving interventions, including high-intensity focused ultrasound (HIFU), underscoring the potential value of Kishi classification in precision treatment planning (15).

Although MRI-based classification has shown promise for disease stratification, its role in predicting outcomes after UAE remains poorly understood. To date, most studies evaluating UAE for AM have focused on overall treatment efficacy without accounting for disease heterogeneity across imaging subtypes (16). Whether patients with different Kishi subtypes experience distinct clinical responses, symptom improvement, recurrence patterns, and long-term outcomes after UAE has not been comprehensively investigated. Addressing this knowledge gap is essential for optimizing patient selection and developing individualized treatment strategies.

Therefore, the present study aimed to evaluate the clinical efficacy and long-term outcomes of UAE in patients with AM according to the Kishi classification. We further sought to determine whether Kishi subtype is associated with treatment response and postoperative recurrence. We hypothesized that MRI-defined Kishi subtypes represent distinct phenotypes with differential therapeutic responses to UAE and may serve as an imaging biomarker for prognostic stratification and personalized management of adenomyosis.

## Materials and methods

### Study design and ethical approval

This retrospective cohort study was conducted at Guangdong Second Provincial General Hospital and complied with the principles of the Declaration of Helsinki. The study protocol was approved by the Institutional Review Board of Guangdong Second Provincial General Hospital (No. 2024-KY-KZ-390-02). Given the retrospective nature of the study, the requirement for informed consent was waived. This study was designed and reported in accordance with the Strengthening the Reporting of Observational Studies in Epidemiology (STROBE) guidelines for observational cohort studies.

### Study population

We retrospectively reviewed consecutive patients with adenomyosis (AM) who underwent uterine artery embolization (UAE) between January 2015 and March 2021. A total of 634 patients were screened, and 467 eligible patients were included in the final analysis according to predefined inclusion and exclusion criteria.

Eligible patients met the following criteria: (1) age ≥18 years; (2) diagnosis of AM confirmed by clinical manifestations and magnetic resonance imaging (MRI); (3) availability of high-quality preprocedural MRI images suitable for Kishi classification; (4) treatment with UAE; and (5) desire for uterine preservation without future fertility plans. Patients with incomplete clinical or follow-up data were excluded.

The final cohort consisted of 118 patients with type I AM, 50 with type II, 17 with type III, and 282 with type IV disease according to the Kishi classification.

### Diagnosis and MRI-based Kishi classification

The diagnosis of AM was established based on clinical symptoms and MRI findings. Typical clinical manifestations included dysmenorrhea, heavy menstrual bleeding, chronic pelvic pain, and dyspareunia. MRI diagnostic criteria included a junctional zone thickness >12 mm and/or the presence of high-signal-intensity foci within the myometrium on T2-weighted imaging.

All MRI examinations were independently reviewed by a radiologist and a gynecologist with over 10-years’ experience who were blinded to clinical outcomes. Discrepancies were resolved by consensus with a third senior reviewer.

According to the MRI-based Kishi classification, AM was categorized as follows:

- Type I (intrinsic): lesions originating from the endometrium and junctional zone and extending into the inner myometrium.
- Type II (extrinsic): lesions involving the outer myometrium adjacent to the serosal surface while preserving the junctional zone.
- Type III (intramural): lesions confined to the myometrium without predominant involvement of either the endometrial or serosal side.
- Type IV (indeterminate): lesions that could not be classified into types I–III.

### MRI acquisition protocol

All patients underwent pelvic magnetic resonance imaging (MRI) within 1 month before UAE. MRI examinations were performed using a 3.0-T scanner (Siemens Healthineers, Erlangen, Germany) with a phased-array pelvic coil according to the institutional imaging protocol.

The MRI protocol included axial, sagittal, and coronal T2-weighted imaging (T2WI), axial T1-weighted imaging (T1WI), and fat-suppressed T1WI sequences. Typical imaging parameters were as follows: repetition time (TR), 3,000–5,000 ms; echo time (TE), 80–120 ms; slice thickness, 4–5 mm; interslice gap, 0.5–1.0 mm; field of view (FOV), 220–260 mm; and matrix size, 256 × 256. Contrast-enhanced imaging was performed when clinically indicated.

MRI examinations were reviewed to assess lesion distribution, junctional zone morphology, uterine volume, concomitant uterine fibroids, ovarian endometrioma, and Kishi subtype classification.

### Sensitivity analysis

Given the unequal distribution of Kishi subtypes in the study cohort, sensitivity analyses were performed to evaluate the robustness of the primary findings. Clinical efficacy and recurrence outcomes were reanalyzed after adjustment for potential confounding factors, including age, uterine volume, hemoglobin level, serum CA125 level, concomitant ovarian endometrioma, and concomitant uterine fibroids.

In addition, subgroup analyses were conducted according to the presence or absence of ovarian endometrioma and uterine fibroids to explore whether these coexisting conditions modified the association between Kishi subtype and clinical outcomes following UAE.

### UAE procedure

All UAE procedures were performed by experienced interventional radiologists using a standardized protocol. Under local anesthesia, bilateral uterine artery catheterization was achieved via a transfemoral approach using the Seldinger technique under digital subtraction angiography guidance. Embolization was performed using calibrated trisacryl gelatin microspheres (Embosphere®, 300–500 μm; Biosphere Medical, Paris, France) until near-complete stasis of uterine arterial flow was achieved bilaterally. Patients were routinely monitored after the procedure and discharged according to institutional protocols.

### Data collection

Baseline demographic and clinical characteristics were extracted from electronic medical records, including age, number of miscarriages, duration of dysmenorrhea, uterine volume, hemoglobin level, serum cancer antigen 125 (CA125) level, concomitant ovarian endometrioma (OE), concomitant uterine fibroids (UFs), and Kishi subtype. The follow-up information was recorded by treating gynecologists or trained clinical staff and was retrospectively extracted for this study using a predefined data collection form. Radiologists were not involved in follow-up assessment and were blinded to follow-up outcomes during MRI interpretation.

Uterine volume was calculated using the ellipsoid formula:

Uterine volume = D1×D2×D3×π÷6 , where D₁, D₂, and D₃ represent the longitudinal, transverse, and anteroposterior uterine diameters, respectively.

Patient-reported outcomes were assessed using the Numerical Rating Scale (NRS), Symptom Severity Scale (SSS), and Health-Related Quality of Life (HRQOL) scale derived from the validated Uterine Fibroid Symptom and Quality of Life (UFS-QOL) questionnaire. Scores were collected before UAE and at 3 months after treatment. NRS scores ranged from 0 to 10, with higher scores indicating more severe pain. SSS and HRQOL scores were transformed to a standardized 0–100 scale; higher SSS scores reflected greater symptom burden, whereas higher HRQOL scores indicated better quality of life.

This study focused on clinical efficacy outcomes, including symptom improvement and long-term recurrence, according to MRI-based adenomyosis subtype classification. Procedural safety outcomes, including postembolization syndrome, infection, amenorrhea, readmission, and detailed reintervention events, were not predefined data elements in the retrospective database and were therefore not included in the present analysis.

### Follow-up and outcome assessment

Clinical follow-up was performed through outpatient visits, inpatient records, and structured telephone interviews. Symptom assessment was conducted 3 months after UAE using NRS, SSS, and HRQOL scores.

#### Primary outcome

The primary outcome was clinical response at 3 month and 5 years after UAE. Clinical response was defined as a reduction of more than 50% in SSS score compared with baseline:

SSS relief rate = (SSS score before treatment–SSS score after treatment)/SSS score before treatment × 100%.

Patients with an SSS reduction rate >50% were classified as responders; all others were classified as non-responders.

#### Secondary outcomes

Secondary outcomes included dysmenorrhea relief and recurrence after UAE.

Dysmenorrhea relief was categorized as:

- Complete relief: NRS score reduced to 0.
- Marked relief: NRS reduction ≥2 points.
- Partial relief: NRS reduction of 1 point.
- No relief: unchanged NRS score.

The dysmenorrhea relief rate was calculated as the proportion of patients achieving complete, marked, or partial relief.

#### Recurrence

Recurrence analysis was restricted to patients who achieved an initial clinical response at 3 months after UAE. Recurrence was defined as a decline in symptom control resulting in an SSS reduction rate ≤50% during follow-up. Patients who subsequently underwent additional interventions for recurrent AM, including hysterectomy, levonorgestrel-releasing intrauterine system implantation, endometrial ablation, or dienogest therapy, were also classified as having experienced recurrence regardless of symptom scores.

### Statistical analysis

Statistical analyses were performed using SPSS version 27.0 (IBM Corp., Armonk, NY, United States) and GraphPad Prism version 9.5 (GraphPad Software, San Diego, CA, United States). Continuous variables were assessed for normality using the Kolmogorov–Smirnov test. Normally distributed variables are presented as mean ± standard deviation and were compared using Student’s *t*-test or one-way analysis of variance (ANOVA) with Tukey’s *post hoc* correction. Non-normally distributed variables are reported as median (interquartile range) and were compared using the Mann–Whitney *U* test or Kruskal–Wallis’s test, followed by pairwise comparisons with Bonferroni adjustment when appropriate. Categorical variables are presented as frequencies and percentages and were compared using the *χ^2^* test or Fisher’s exact test.

Univariable and multivariable logistic regression analyses were performed to identify independent predictors of clinical response and 5-year recurrence. Variables with *p* < 0.10 in univariable analyses were entered into multivariable models. Results are reported as odds ratios (ORs) with 95% confidence intervals (CIs). All statistical tests were two-sided, and a *p* value <0.05 was considered statistically significant.

## Results

### Patient characteristics according to Kishi classification

**Figure 1:**
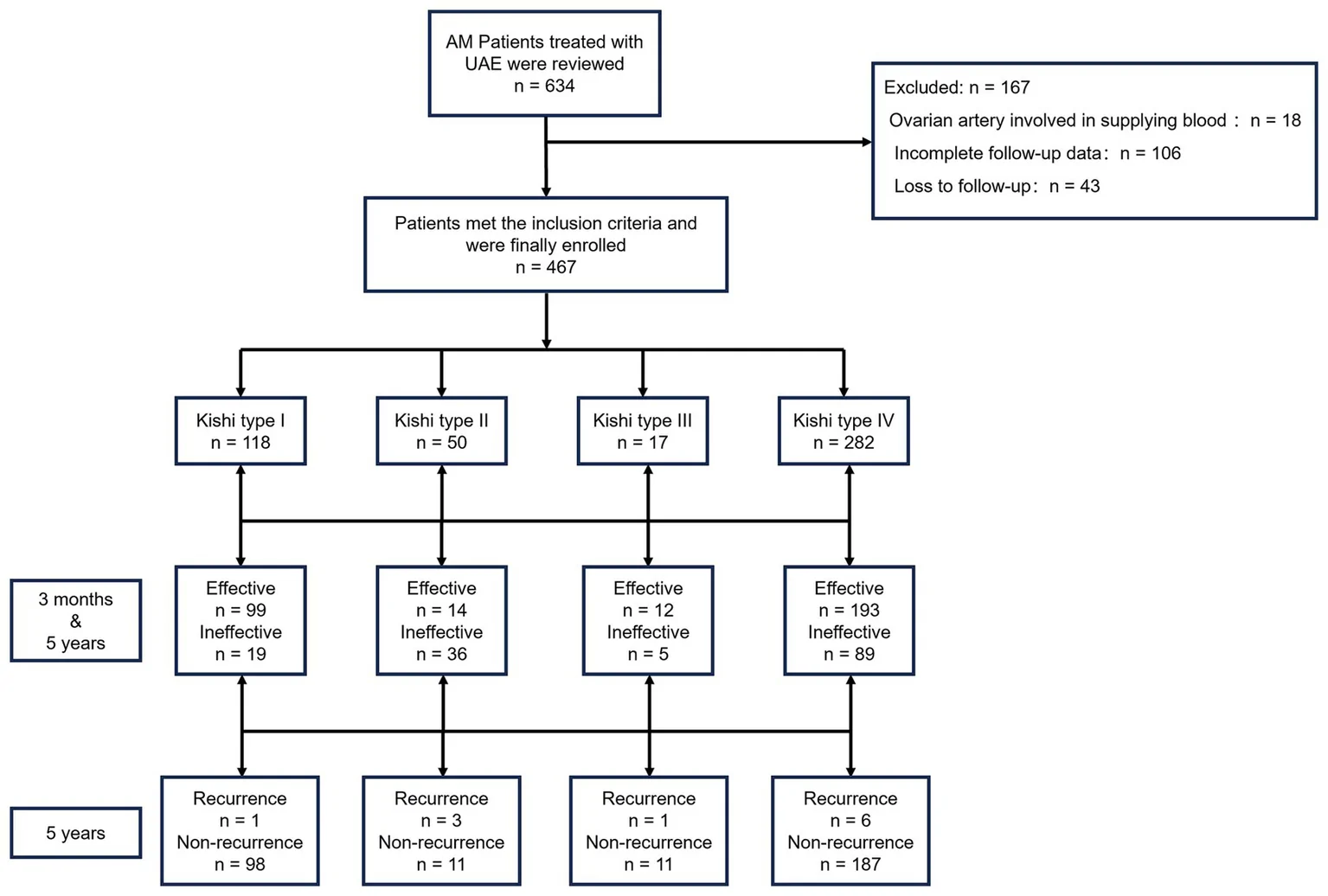
Flowchart of patient selection.

Among 634 patients screened, 467 met the eligibility criteria and were included in the final analysis. According to the Kishi classification, 118 patients (25.3%) were classified as type I, 50 (10.7%) as type II, 17 (3.6%) as type III, and 282 (60.4%) as type IV (Figure 1).

Baseline characteristics are summarized in Table 1. Significant differences were observed among the four subtypes with respect to age, number of miscarriages, uterine volume, hemoglobin level, serum CA125 level, and the prevalence of concomitant ovarian endometrioma (all *p* < 0.05). In general, type I patients were older and had lower CA125 levels and lower rates of concomitant ovarian endometrioma than type II patients. Type II patients were younger and exhibited the highest prevalence of ovarian endometrioma. No significant differences were observed in dysmenorrhea duration or the prevalence of concomitant uterine fibroids among the four groups (*p* > 0.05).

**Table 1:** Comparisons of general clinical baseline data.

Clinical indicators	Type I (*n* = 118)	Type II (*n* = 50)	Type III (*n* = 17)	Type IV (*n* = 282)
Age (years)	41.20 ± 4.56	39.40 ± 5.14^a^	41.71 ± 3.90	40.92 ± 4.96^b^
Number of miscarriages (times)	1 (0, 8)	1 (0, 7)^a^	1 (0, 7)	1 (0, 10)^bb^
Uterine volume (cm^3^)	242.35 (84.90, 1024.40)	231.65 (66.70, 780.40)	219.60 (68.60, 520.10)	279.20 (58.60, 1477.80)^aabbc^
Dysmenorrhea duration (years)	3 (1, 25)	5 (5, 20)	3 (1, 14)	4 (1, 29)
Hemoglobin (g/L)	99.91 ± 25.48	115.54 ± 20.10^a^	104.06 ± 24.36	102.82 ± 22.66^b^
CA125 (U/mL)	55.15 (5.87, 933.80)	82.62 (14.19, 795.50)^a^	62.24 (10.53, 448.88)	83.81 (10.67, 1006.00)^a^
Comorbid UFs (*n*, %)	31 (26.27%)	17 (34.00%)	4 (23.53%)	94 (33.33%)
Comorbid OE (*n*, %)	12 (10.17%)	26 (52.00%)^aa^	3 (17.65%)^b^	43 (15.25%)^bb^

CA125, cancer antigen 125. Measurement data of normal distribution were expressed as mean ± SD. One-way ANOVA was used for comparisons among multiple groups, followed by Tukey’s multiple comparison tests. Measurement data of non-normal distribution were represented by quartiles, i.e., median value (minimum value, maximum value). The Kruskal-Wallis test was used for comparisons among multiple groups, and the Dunn’s multiple comparison test was used for post-hoc tests. The measurement data were expressed by the number of cases and percentages, and the chi-square test was used for comparisons between groups. Compared with type I: ^a^represented *p* < 0.05, ^aa^represented *p* < 0.01; compared with type II: ^b^represented *p* < 0.05, ^bb^represented *p* < 0.01; compared with type III: ^c^represented *p* < 0.05. Differences were considered to be significant at *p* < 0.05.

### Symptom severity and quality-of-life outcomes after UAE

Changes in Symptom Severity Scale (SSS) and Health-Related Quality of Life (HRQOL) scores are presented in Table 2.

**Table 2:** Changes of SSS and HRQOL scores in AM patients with different Kishi types and comparisons between groups.

Clinical indicators	Type I (*n* = 118)	Type II (*n* = 50)	Type III (*n* = 17)	Type IV (*n* = 282)
SSS score (%)				
Pre-operation	53.13 (18.75, 90.63)	31.25 (6.25, 90.63)^aa^	46.88 (34.38, 90.63)^bb^	53.13 (3.13, 96.88)^bb^
3-months post operation	9.38 (0.00, 65.63)^dd^	21.88 (0.00, 81.25)^aadd^	14.65 (2.72, 21.88)^add^	15.63 (0.00, 100.00)^aadd^
HRQOL score (%)				
Pre-operation	42.80 ± 22.04	49.31 ± 21.53	44.78 ± 15.26	44.84 ± 23.44
3-months post operation	79.63 ± 16.18^dd^	69.35 ± 22.58^add^	78.58 ± 18.21^dd^	75.96 ± 19.89^bdd^

SSS, Symptom Severity Scale; HRQOL, Health-related Quality of Life Scale. Measurement data of normal distribution were expressed as mean ± SD. One-way ANOVA was used for comparisons among multiple groups, followed by Tukey’s multiple comparison tests. Measurement data of non-normal distribution were represented by quartiles, i.e., median value (minimum value, maximum value). The Kruskal-Wallis test was used for comparisons among multiple groups, and the Dunn’s multiple comparison test was used for post-hoc tests. Compared with type I: ^a^represented *p* < 0.05, ^aa^represented *p* < 0.01; compared with type II: ^b^represented *p* < 0.05, ^bb^represented *p* < 0.01; compared with pre-operation: ^dd^represented *p* < 0.01. Differences were considered to be significant at *p* < 0.05.

Before UAE, type II patients demonstrated significantly lower SSS scores than patients with the other Kishi subtypes (all *p* < 0.05), whereas baseline HRQOL scores were comparable across groups. At 3 months after UAE, SSS scores decreased significantly and HRQOL scores improved significantly in all subtypes compared with baseline (all *p* < 0.01).

Postoperatively, type I patients had the lowest SSS scores among all subtypes, indicating the greatest symptom improvement. In contrast, type II patients had significantly lower clinical response rates than those with types I, III and IV (*p* < 0.05). Type II patients exhibited significantly lower HRQOL scores than type I and type III patients (*p* < 0.05), suggesting less favorable quality-of-life recovery following UAE.

### Improvement in dysmenorrhea following UAE

Baseline Numerical Rating Scale (NRS) scores were similar among the four Kishi subtypes (Table 3). Three months after UAE, NRS scores decreased significantly in all groups (all *p* < 0.01).

**Table 3:** Changes of NRS score and dysmenorrhea relief in AM patients with different Kishi types and comparisons between groups.

Clinical indicators	Type I (*n* = 118)	Type II (*n* = 50)	Type III (*n* = 17)	Type IV (*n* = 282)
NRS score (%)				
Pre-operation	5 (1, 10)	6 (2, 10)	5 (2, 10)	7 (1, 10)
3-months post operation	0 (0, 10)^dd^	2 (0, 10)^aadd^	1 (0, 5)^aadd^	1 (0, 10)^abbdd^
Dysmenorrhea relief (%)				
Remission	110 (93.22%)	39 (78.00%)^aa^	17 (100%)	233 (82.62%)^aa^
Non-remission	8 (6.78%)	11 (22.00%)^aa^	2 (0)	49 (17.38%)^aa^

NRS, Numeric Rating Scale. Measurement data of non-normal distribution were represented by quartiles, i.e., median value (minimum value, maximum value). The Kruskal-Wallis test was used for comparisons among multiple groups, and the Dunn’s multiple comparison test was used for post-hoc tests. The measurement data were expressed by the number of cases and percentages, and the chi-square test was used for comparisons between groups. Compared with type I: ^a^represented *p* < 0.05, ^aa^represented *p* < 0.01; compared with type II: ^bb^represented *p* < 0.01; compared with pre-operation: ^dd^represented *p* < 0.01. Differences were considered to be significant at *p* < 0.05.

Type I patients achieved the lowest postoperative NRS scores, whereas type II patients reported significantly higher residual pain levels than type IV patients (*p* < 0.05). Dysmenorrhea relief rates were 93.2% (110/118) in type I, 78.0% (39/50) in type II, 88.2% (15/17) in type III, and 82.6% (233/282) in type IV patients. The relief rate was significantly higher in type I and III than in type II and type IV patients (both *p* < 0.05), whereas no significant differences were observed among types I and III.

### Clinical response according to Kishi classification

Clinical response rates at 3 months after UAE were shown in Table 4. Overall, 318 patients (68.1%) achieved a clinical response.

**Table 4:** Evaluation of the therapeutic effect of AM patients with different Kishi types at 5 years post operation.

Clinical indicators	Type I (*n* = 118)	Type II (*n* = 50)	Type III (*n* = 17)	Type IV (*n* = 282)
Therapeutic effect (*n*, %)				
Effective	99 (83.90%)	14 (28.00%)^aa^	12 (70.59%)^bb^	193 (68.44%)^aabb^
Ineffective	19 (16.10%)	36 (72.00%)^aa^	5 (29.41%)^bb^	89 (31.56%)^aabb^

The measurement data were expressed by the number of cases and percentages, and the chi-square test was used for comparisons between groups. Compared with type I: ^aa^represented *p* < 0.01; compared with type II: ^bb^represented *p* < 0.01. Differences were considered to be significant at *p* < 0.05.

Response rates differed significantly among Kishi subtypes (*p* < 0.001), with the highest response observed in type I patients (83.9%), followed by type III (70.6%), type IV (68.4%), and type II patients (28.0%). Compared with type II disease, both type III and type IV disease demonstrated significantly higher response rates (*p* < 0.01). Type I patients achieved significantly better outcomes than patients with type II and type IV disease (*p* < 0.01).

### Factors associated with clinical response after UAE

To identify predictors of treatment response, patients were categorized into responder (*n* = 318) and non-responder (*n* = 149) groups (Table 5).

**Table 5:** The relationship between clinical baseline data and the therapeutic effect of UAE in AM patients.

Clinical indicators	Effective group (*n* = 318)	Ineffective group (*n* = 149)	*p*
Age (years)	41.32 ± 4.68	39.87 ± 5.10	0.002
number of miscarriages (times)	1 (0, 10)	1 (0, 7)	0.901
Uterine volume (cm^3^)	260.45 (58.60, 1477.80)	257.60 (66.70, 1254.10)	0.633
Dysmenorrhea duration (years)	4 (1, 29)	4 (3, 25)	0.450
Hemoglobin (g/L)	101.51 ± 23.98	107.72 ± 20.07	0.008
CA125 (U/mL)	73.01 (6.67, 933.80)	78.56 (5.87, 1006.00)	0.883
Comorbid UFs (*n*, %)	97 (30.50%)	49 (32.89%)	0.605
Comorbid OE (*n*, %)	41 (12.89%)	43 (28.86%)	<0.001
Kishi classification			<0.001
Type I	99 (31.13%)	19 (12.75%)	
Type II	14 (4.40%)	36 (24.16%)	
Type III	12 (3.77%)	5 (3.36%)	
Type IV	193 (60.69%)	89 (59.73%)	

CA125, cancer antigen 125. Measurement data of normal distribution were expressed as mean ± SD. The independent sample t test was used for comparisons between two groups. Measurement data of non-normal distribution were expressed as quartiles, i.e., median (minimum, maximum). The Mann–Whitney U test was used for comparisons between two groups. The measurement data were expressed by the number of cases and percentages, and the chi-square test was used for comparisons between groups. *p* < 0.05 indicated that the difference was statistically significant.

Responders were significantly older and had lower hemoglobin levels and lower rates of concomitant ovarian endometrioma than non-responders (all *p* < 0.05). In addition, the distribution of Kishi subtypes differed significantly between groups, with a higher proportion of type I and a lower proportion of type II disease among responders (*p* < 0.001).

In univariable logistic regression analysis (Table **6**), age, hemoglobin level, concomitant ovarian endometrioma, and Kishi classification were significantly associated with clinical response after UAE. After multivariable adjustment, age (OR, 1.059; 95% CI, 1.012–1.107; *p* = 0.013), concomitant ovarian endometrioma (OR, 0.504; 95% CI, 0.292–0.870; *p* = 0.014), and Kishi classification remained independent predictors of treatment response. Compared with other subtypes, type I disease was independently associated with a higher likelihood of clinical response (OR, 1.940; 95% CI, 1.093–3.443; *p* = 0.024), whereas type II disease was associated with a significantly lower likelihood of response (OR, 0.268; 95% CI, 0.136–0.531; *p* < 0.001).

**Table 6:** Logistic regression model analysis of influencing factors on clinical efficacy of AM patients.

Univariate analysis				Multivariate analysis		
Clinical indicators	*p*	OR	95% CI	*p*	OR	95% CI
Age (years)	0.003	1.064	1.022–1.109	0.015	1.055	1.010–1.102
Number of miscarriages (times)	0.977	0.998	0.880–1.130	—	—	—
Uterine volume (cm^3^)	0.426	1.000	0.999–1.002	—	—	—
Dysmenorrhea duration (years)	0.132	0.968	0.928–1.010	—	—	—
Hemoglobin (g/L)	0.008	0.989	0.980–0.997	0.135	0.993	0.984–1.002
CA125 (U/mL)	0.598	1.000	0.998–1.001	—	—	—
Comorbid UFs (*n*, %)	0.605	0.896	0.590–1.359	—	—	—
Comorbid OE (*n*, %)	< 0.001	0.365	0.225–0.591	0.022	0.536	0.314–0.916
Kishi classification						
Type I	0.002	2.403	1.384–4.170	0.004	2.276	1.302–3.977
Type II	< 0.001	0.179	0.092–0.349	< 0.001	0.252	0.125–0.509
Type III	0.853	1.107	0.378–3.236	0.886	1.083	0.365–3.211

CA125, cancer antigen 125. Logistic regression was used to evaluate the influencing factors for clinical efficacy in AM patients. The Reference Category for the Kishi classification was set to Last, i.e., the last classification was set as a reference. *p* < 0.05 indicated that the difference was statistically significant.

### Long-term recurrence according to Kishi classification

Among the 318 patients who achieved an initial clinical response, 5-year recurrence outcomes were evaluated (Table 7).

**Table 7:** Evaluation of recurrence situation within 5 years after UAE surgery in AM patients with different Kishi types.

Recurrence situation [*n*, %]	Type I (*n* = 99)	Type II (*n* = 14)	Type III (*n* = 12)	Type IV (*n* = 193)	Overall
Recurrence	1 (1.0%)	3 (21.4%)^aa^	1 (8.3%)	6 (3.1%)^b^	11 (3.5%)
Non-recurrence	98 (99.0%)	11 (78.6%)^aa^	12 (91.7%)	187 (96.9%)^b^	307 (96.6%)
95% CI	0.03–5.5	4.7–50.8	0.2–38.5	1.2–6.7	1.7–6.1

Measurement data were expressed by the number of cases and percentages, and two-sided Fisher’s exact test was used for comparisons between groups. Compared with type I: ^aa^represented *p* < 0.01; compared with type II: ^b^represented *p* < 0.05. Differences were considered to be significant at *p* < 0.05.

The recurrence rates were 1.0% (1/99) for type I, 21.4% (3/14) for type II, 8.3% (1/12) for type III, and 3.1% (6/193) for type IV disease. Type II patients exhibited significantly higher rates of recurrence than type I, type III and type IV patients (all *p* < 0.01).

### Factors associated with 5-year efficacy

Univariable logistic regression analysis identified age, dysmenorrhea duration (years), hemoglobin level, concomitant ovarian endometrioma, and Kishi classification were significantly associated with 5-Year efficacy (Table 8). After multivariable adjustment, age (OR, 1.053; 95% CI, 1.008–1.101; *p* = 0.021), concomitant ovarian endometrioma (OR, 0.361; 95% CI, 0.212–0.614; *p* < 0.001), and Kishi classification remained independent predictors of treatment response. Compared with other subtypes, type I disease was independently associated with a higher likelihood of clinical efficacy (OR, 2.226; 95% CI, 1.269–3.907; *p* = 0.005), whereas type II disease was associated with a significantly lower likelihood of efficacy (OR, 0.269; 95% CI, 0.132–0.547; *p* < 0.001).

**Table 8:** Logistic regression model analysis of factors affecting postoperative recurrence in AM patients within 5 years.

Univariate analysis				Multivariate analysis		
Clinical indicators	*p*	OR	95% CI	*p*	OR	95% CI
Age (years)	0.934	0.998	0.946–1.052	—	—	—
number of miscarriages (times)	0.702	0.970	0.831–1.133	—	—	—
Uterine volume (cm^3^)	0.771	1.000	0.999–1.002	—	—	—
Dysmenorrhea duration (years)	0.823	1.007	0.948–1.069	—	—	—
Hemoglobin (g/L)	0.249	0.994	0.984–1.004	—	—	—
CA125 (U/mL)	0.011	0.997	0.995–0.999	0.016	0.997	0.995–1.000
Comorbid UFs [*n*, %]	0.345	0.778	0.459–1.313	—	—	—
Comorbid OE [*n*, %]	0.935	1.031	0.492–2.160	—	—	—
Kishi classification						
Type I	0.126	1.574	0.880–2.817	0.175	1.518	0.830–2.774
Type II	0.032	0.299	0.099–0.901	0.022	0.254	0.078–0.824
Type III	0.720	0.797	0.173–2.755	0.625	0.732	0.210–2.553

CA125, cancer antigen 125. Logistic regression was used to evaluate the influencing factors of recurrence in AM patients within 5 years after operation. The Reference Category for the Kishi classification was set to Last, i.e., the last classification was set as a reference. *p* < 0.05 indicated that the difference was statistically significant.

## Discussion

In this retrospective cohort study of 467 patients with adenomyosis treated with uterine artery embolization (UAE), we demonstrated that MRI-based Kishi classification was significantly associated with baseline clinical characteristics, short-term symptom improvement, treatment response, and long-term recurrence. The principal findings were threefold. First, distinct Kishi subtypes exhibited different clinical phenotypes, particularly regarding age, uterine volume, serum CA125 level, and the prevalence of concomitant ovarian endometrioma. Second, patients with type I (intrinsic) adenomyosis achieved the most favorable outcomes after UAE, including greater symptom relief, better quality-of-life improvement, and the highest clinical response rate. Third, type II (extrinsic) adenomyosis was independently associated with both poorer treatment response and a substantially higher risk of recurrence during long-term follow-up. Collectively, these findings suggest that Kishi classification may serve as a clinically useful imaging biomarker for treatment stratification and prognostic assessment before UAE.

The observed differences in baseline characteristics among Kishi subtypes further support the concept that adenomyosis represents a heterogeneous disease entity rather than a single pathological condition. Consistent with previous studies, patients with type II adenomyosis were younger and demonstrated a markedly higher prevalence of concomitant ovarian endometrioma than patients with other subtypes (17). Emerging evidence suggests that extrinsic adenomyosis and endometriosis may share common pathogenic mechanisms, including retrograde menstruation, chronic inflammation, and estrogen-dependent tissue invasion. In contrast, intrinsic adenomyosis has been linked more closely to disruption of the endometrial–myometrial interface and uterine injury associated with repeated intrauterine procedures (18). These distinct pathogenetic pathways may explain the differences in clinical presentation observed across Kishi subtypes. Moreover, the higher CA125 levels and larger uterine volumes observed in patients with diffuse or indeterminate (type IV) likely reflect a greater overall disease burden and more extensive myometrial involvement (19, 20).

Adenomyosis frequently coexists with endometriosis, and increasing evidence suggests that these conditions may represent overlapping but distinct estrogen-dependent inflammatory disorders (21). Coexisting endometriosis may contribute to pelvic pain persistence and may influence long-term symptom outcomes after conservative interventions. Recent imaging-based classification studies have suggested that the extent and anatomical distribution of adenomyotic lesions may correlate with symptom severity, highlighting the importance of disease phenotype characterization (22). In the present study, Kishi subtype classification was evaluated as an MRI-based marker of adenomyosis phenotype and its association with UAE outcomes. However, because the study was not specifically designed to investigate endometriosis-associated mechanisms, the independent contribution of coexisting endometriosis to long-term outcomes remains uncertain. Future prospective studies integrating detailed assessment of endometriosis phenotype, adenomyosis distribution, and longitudinal symptom trajectories are warranted.

One of the most clinically relevant findings of the present study is the substantial heterogeneity in treatment response according to Kishi subtype. Although UAE resulted in significant improvements in symptom severity, dysmenorrhea, and health-related quality of life across all subgroups, patients with type I adenomyosis consistently achieved the greatest benefit. At 3 months after UAE, type I patients demonstrated the lowest SSS and NRS scores, the highest dysmenorrhea relief rate, and the highest overall clinical response rate. These findings are biologically plausible because intrinsic adenomyotic lesions are predominantly located within the inner myometrium and are largely supplied by branches of the uterine artery, which represent the primary target of embolization. Consequently, ischemic necrosis induced by UAE may be complete and more durable in this subtype. Besides, although MRI provides superior anatomical characterization and was selected as the imaging modality for establishing our classification system, it is not intended as a universal diagnostic tool for all patients with adenomyosis. In clinical practice, TVUS remains the primary imaging modality, while MRI may serve as a complementary examination for complex cases, preoperative assessment, and detailed lesion characterization.

In contrast, patients with type II adenomyosis experienced significantly less symptom improvement and substantially lower clinical response rates. Several mechanisms may explain this observation. Extrinsic adenomyosis is frequently associated with pelvic endometriosis, deep infiltrating endometriosis, and ovarian endometrioma, conditions characterized by complex vascular networks extending beyond the uterus (23). Previous imaging and surgical studies have demonstrated that extrinsic lesions often involve the outer myometrium and serosal surface and may receive blood supply from extrauterine collateral vessels (24). As a result, embolization of the uterine arteries alone may not completely eliminate lesion perfusion, potentially limiting treatment efficacy and predisposing patients to symptom persistence (25). Similar subtype-dependent differences in therapeutic response have also been reported following high-intensity focused ultrasound (HIFU), where intrinsic adenomyosis generally exhibits more favorable outcomes than extrinsic disease (26). Taken together, these observations suggest that disease biology, rather than procedural factors alone, may substantially influence treatment success in uterus-preserving therapies. Nevertheless, not all studies support a uniform interpretation of extrinsic adenomyosis or its therapeutic implications. The definition of extrinsic adenomyosis remains debated, with some authors considering it a distinct subtype related to endometriosis and outer myometrial invasion, whereas others regard it as part of a continuum between adenomyosis and deep endometriosis or as a consequence of differences in imaging and pathological criteria. Similarly, the efficacy of uterine artery embolization in adenomyosis is heterogeneous. While symptom improvement has been reported in selected patients, other studies have suggested that outcomes may differ according to lesion distribution, vascularity, coexistence of leiomyomas, and the presence of extrinsic disease. These contrasting findings indicate that adenomyosis should not be regarded as a single homogeneous condition and support the need for more precise imaging-based phenotyping.

Another important finding is the prognostic value of Kishi classification for long-term recurrence. Among patients who initially responded to UAE, Type II and III patients experienced a higher rate of 5-year recurrence following UAE than type I and type IV patients. Furthermore, after correction of the recurrence data, type II adenomyosis showed the highest crude recurrence rate. However, this finding should be interpreted cautiously because the number of recurrence events was small, particularly in types II and III, and the corresponding confidence intervals were wide. Therefore, the observed subtype-specific recurrence pattern should be considered exploratory and requires confirmation in larger prospective cohorts with longer and standardized follow-up.

The identification of Kishi classification as a predictor of both treatment response and recurrence has important clinical implications. Currently, patient selection for UAE is primarily based on symptom severity, uterine preservation preference, and imaging confirmation of adenomyosis (27, 28). Our findings suggest that MRI-based subtype assessment should be incorporated into the preprocedural evaluation process. Patients with type I adenomyosis appear to be particularly suitable candidates for UAE and may expect favorable short- and long-term outcomes. Conversely, patients with type II disease should be counseled regarding the increased risk of treatment failure and recurrence. For these patients, combined therapeutic approaches, such as adjunctive hormonal therapy, management of coexisting endometriosis, or multimodal treatment strategies, may warrant further investigation.

This study has several strengths. To our knowledge, it represents one of the largest cohorts evaluating UAE outcomes according to the Kishi classification and is among the first to systematically assess both short-term efficacy and long-term recurrence across MRI-defined adenomyosis subtypes. The relatively large sample size, standardized UAE protocol, and comprehensive follow-up allowed us to characterize clinically meaningful differences among Kishi subtypes and identify independent prognostic factors.

Several limitations should also be acknowledged. First, the retrospective single-center design may introduce selection bias and limit causal inference. From a disease management perspective, future research should focus on integrating imaging phenotypes with clinical symptoms, reproductive plans, biomarkers, and treatment preferences to guide individualized management. Phenotype-specific studies may help clarify which patients are more likely to benefit from medical therapy, conservative surgery, high-intensity focused ultrasound, uterine artery embolization, or assisted reproductive strategies. Ultimately, a validated classification system may contribute to risk stratification, treatment selection, follow-up planning, and multidisciplinary decision-making in adenomyosis. Second, the relatively small number of patients with type III adenomyosis reduced statistical power for comparisons involving this subgroup. Besides, because the present study was retrospective and conducted in a limited clinical setting, the reproducibility and clinical utility of this classification should be confirmed in independent, multicenter cohorts with standardized imaging protocols and blinded image interpretation. Future studies should also determine whether this classification can predict clinically relevant outcomes, including symptom severity, fertility outcome, recurrence, and response to different treatments. Third, recurrence was primarily assessed using symptom-based criteria rather than routine MRI confirmation, which may have resulted in underestimation or misclassification of disease recurrence. Finally, potential confounding factors related to postoperative medical therapy and the severity of coexisting endometriosis could not be fully accounted for.

In conclusion, MRI-based Kishi classification is independently associated with both clinical response and long-term recurrence after UAE in patients with adenomyosis. Type I adenomyosis is associated with the most favorable therapeutic outcomes, whereas type II disease demonstrates substantially lower response rates and a higher risk of recurrence. These findings support the integration of Kishi classification into preprocedural evaluation and suggest that MRI-based phenotyping may facilitate individualized treatment selection and prognostic stratification in patients undergoing UAE.

## Data Availability

The original contributions presented in the study are included in the article/supplementary material, further inquiries can be directed to the corresponding author.
